# Quantitative CT comparison between COVID-19 and mycoplasma pneumonia suspected as COVID-19: a longitudinal study

**DOI:** 10.1186/s12880-022-00750-4

**Published:** 2022-02-06

**Authors:** Junzhong Liu, Yuzhen Wang, Guanghui He, Xinhua Wang, Minfeng Sun

**Affiliations:** 1 Department of Radiology, Weifang No. 2 People’s Hospital, The Second Affiliated Hospital of Weifang Medical University, Weifang City, Shandong Province China; 2 Department of Interventional Radiology, Weifang No. 2 People’s Hospital, The Second Affiliated Hospital of Weifang Medical University, Weifang City, Shandong Province China; 3 Department of Medical Imaging, Weifang No. 2 People’s Hospital, The Second Affiliated Hospital of Weifang Medical University, 7 Yuanxiao Street, Weifang City, 261041 Shandong Province People’s Republic of China

**Keywords:** COVID-19, Mycoplasma pneumonia, Quantitative CT

## Abstract

**Objective:**

The purpose of this study was to compare imaging features between COVID-19 and mycoplasma pneumonia (MP).

**Materials and methods:**

The data of patients with mild COVID-19 and MP who underwent chest computed tomography (CT) examination from February 1, 2020 to April 17, 2020 were retrospectively analyzed. The Pneumonia-CT-LKM-PP model based on a deep learning algorithm was used to automatically quantify the number, volume, and involved lobes of pulmonary lesions, and longitudinal changes in quantitative parameters were assessed in three CT follow-ups.

**Results:**

A total of 10 patients with mild COVID-19 and 13 patients with MP were included in this study. There was no difference in lymphocyte counts at baseline between the two groups (1.43 ± 0.45 vs. 1.44 ± 0.50, *p* = 0.279). C-reactive protein levels were significantly higher in MP group than in COVID-19 group (*p* < 0.05). The number, volume, and involved lobes of pulmonary lesions reached a peak in 7–14 days in the COVID-19 group, but there was no peak or declining trend over time in the MP group (*p* < 0.05).

**Conclusion:**

Based on the longitudinal changes of quantitative CT, pulmonary lesions peaked at 7–14 days in patients with COVID-19, and this may be useful to distinguish COVID-19 from MP and evaluate curative effects and prognosis.

## Background

Coronavirus Disease-19 (COVID-19) is a highly infectious lung disease caused by a novel coronavirus. On February 11, 2020, the International Virus Classification Committee officially named the novel coronavirus as "SARS-CoV-2." The World Health Organization termed the new coronavirus pneumonia as COVID-19 [1].

The diagnosis of COVID-19 depends on nucleic acid detection, but the sensitivity is not high, and there are many false negatives. High-resolution computed tomography (HRCT) screening can detect early lung changes in patients with COVID-19 and provide more diagnostic information [2]. Although it is possible for a radiologist to diagnose COVID-19 through CT manifestations of bilateral ground glass opacity (GGO) and/or consolidation [3], a diagnosis of COVID-19 based on imaging may be incorrect because many other diseases can exhibit similar patterns. The CT features of mycoplasma pneumonia (MP) in adults are often similar to those of viral interstitial pneumonia [4–7]. In view of the different infectivity and treatments, it is crucial to accurately differentiate COVID-19 patients who need to be isolated and treated as soon as possible from MP patients.

There have been reports of using artificial intelligence such as deep learning methods for quantitative analysis and diagnosing COVID-19 [8–10]. However, there are no reports in the literature of misdiagnosis of COVID-19 based on CT imaging. In this study, we longitudinally compared the quantitative CT features of 10 patients diagnosed as mild COVID-19 and 13 patients suspected to have COVID-19 but ultimately diagnosed as MP.

## Materials and methods

This retrospective study was approved by the Hospital Ethics Review Committee. The request for personal informed consent was waived.

### Participants

The following inclusion and exclusion criteria were used to screen for patients with suspected COVID-19 who entered the isolation ward of the hospital from February 1, 2020 to April 17, 2020.

The inclusion criteria were: (1) mild COVID-19 illness with a positive real-time reverse-transcriptase polymerase chain reaction (rRT-PCR) test; (2) MP patients with radiographically suspected COVID-19 but negative rRT-PCR result; (3) abnormal initial CT examination, followed by at least three additional CT examinations including follow-up at least 30 days after onset. Complete picture archiving and communication system data were available. The exclusion criteria were: (1) patients who were not in our hospital for the first CT examination, (2) incomplete follow-up data, and/or (3) other types of pneumonia.

All patients in isolation wards underwent nasopharyngeal rRT-PCR testing at least twice. MP was diagnosed based on serum specific IgM antibody positivity.

### CT scanning

The date of initial symptom onset was defined as day 0 of the disease. All patients performed plain CT scans under inspiration using a dual source 128-slice spiral scanner (Siemens, Germany). The CT parameters were as follows: Thorax Routine-Customized protocol, tube voltage 120 kV; automatic tube current; CareDose on; thickness 5 mm; and matrix 512 * 512. The reconstructed image uses a smooth lung core (B70f) at 1.25-mm intervals. Window setting: lung window (width 1500 Hu; level, − 700Hu), mediastinal window (width 400Hu; level 40Hu).

### Variables

**Fig. 1:**
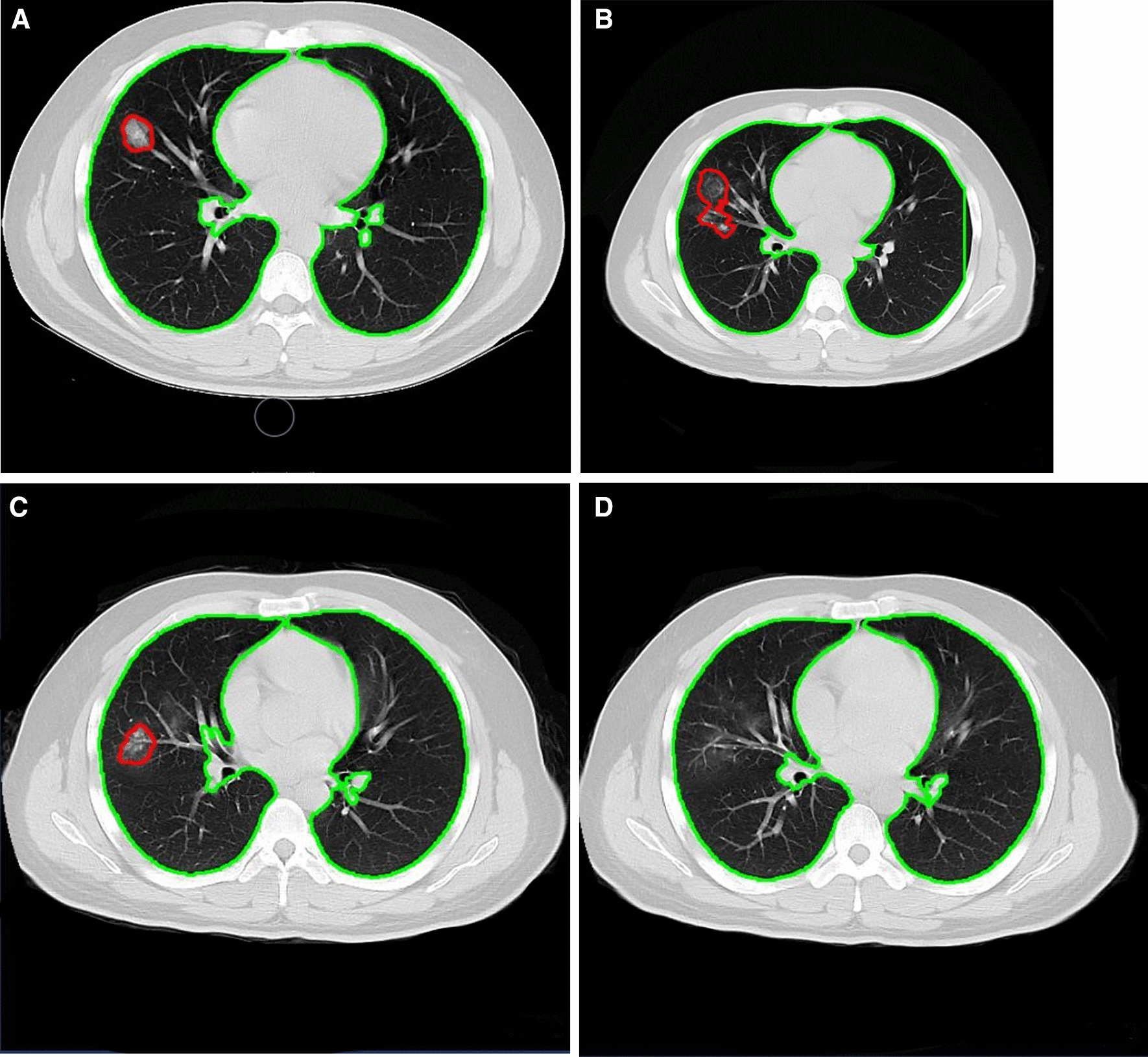
Longitudinal CT in a 29-year-old male with mild COVID-19. **A** The initial CT examination showed a GGO in the middle lobe of the right lung; **B** the lesion enlarged, and new GGO appeared (accounting for 1.32% of the right lung volume); **C** GGO was absorbed compared with the second inspection; **D** The GGO remained at follow-up 30 days later

**Fig. 2:**
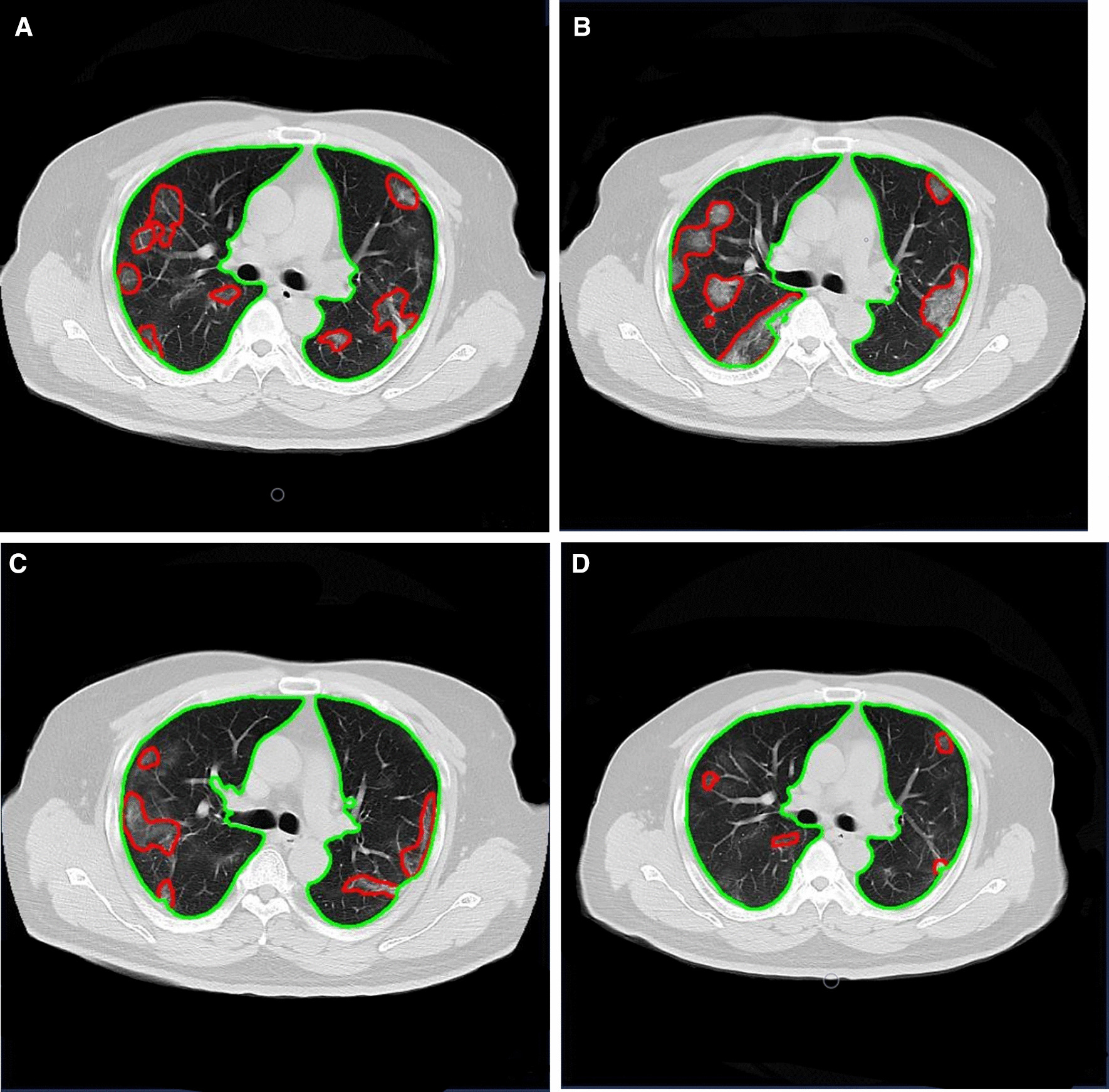
Longitudinal CT in a 41-year-old male with mild COVID-19. **A** Multiple mixed GGO in both lungs under the pleura. **B** The size and number of GGO increased. **C** GGO slightly absorbed compared to the second follow-up. **D** Residual GGO in both lungs were seen in the third follow-up

**Fig. 3:**
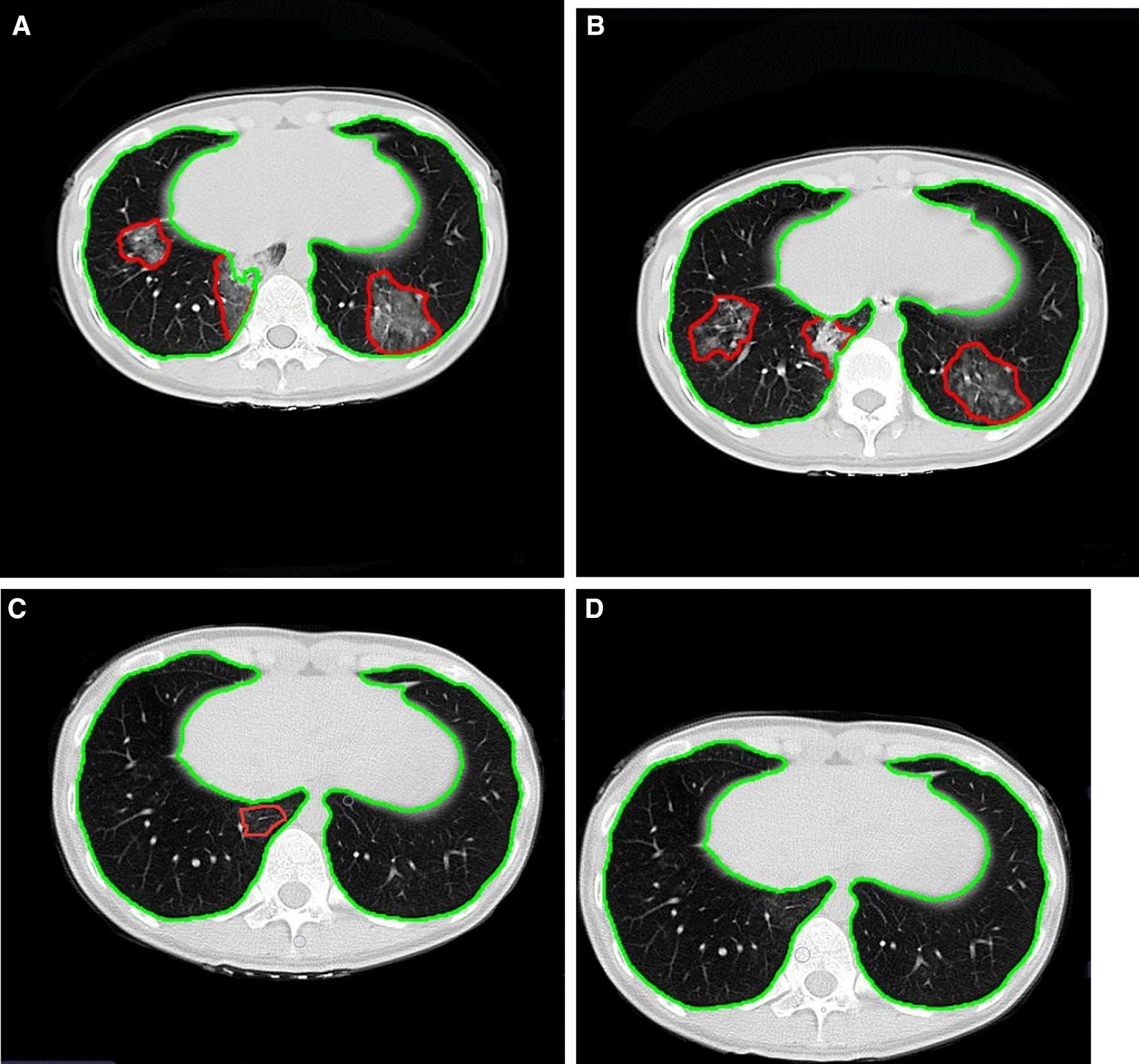
Longitudinal CT in a 24-year-old female hotel attendant exposed to COVID-19 briefly, but confirmed as MP. **A** Multiple GGO and consolidation shadows were observed in the lower lobes of both lungs at the baseline examination. **B** At the first follow-up, the volume of lung lesions was not progressed. **C**, **D** Lung lesion volume gradually decreased and then disappeared during the second and third follow-ups

We used the Pneumonia-CT-LKM-PP model based on a deep learning algorithm to analyze CT images. This model is mainly used to detect and identify pneumonic lesions and can output a full set of quantitative diagnostic indicators such as the number, volume of pneumonic lesions (Figs. 1, 2, 3). The Pneumonia-CT-LKM-PP model can refer to the online version [11]. The related license, which is associated with the Pneumonia-CT-LKM-PP model, is based on the well-known Apache License 2.0. More details can be found at the online link [12].

We examined the locations of lesions on CT images and calculated the score based on all five lobes of both lungs. Each cumulative lobe gets one point, and the total score is five. The scores of all CT images were calculated for each patient to analyze lesion changes over time.

### Statistical analysis

IBM SPSS Statistics 25 (Armonk, NY, USA) was used for all statistical analyses. Categorical variables were compared using Chi-square tests or Fisher's exact tests, and continuous variables were analyzed using t tests. Repeated data was statistically analyzed by analysis of variance. Differences were considered significant at *p* < 0.05. GraphPad Prism 8.0 software (USA) was used to generate graphs.

## Results

### Participants

A total of 10 patients with mild type COVID-19 and 13 patients with suspected COVID-19 who were ultimately diagnosed with MP were enrolled in this study. Ninety percent of patients diagnosed with COVID-19 had a history of exposure to the virus. One case in the MP group had a short exposure but never tested positive. There was no difference in the number of lymphocytes at baseline between the COVID-19 and MP groups (1.4320 ± 0.448 vs. 1.4469 ± 0.500, *p* = 0.279). The C-reactive protein (CRP) index of the MP group was significantly higher than that of the COVID-19 group (*p* < 0.05). In addition, there was no statistical difference between the two groups in parameters such as gender, age, BMI, maximum body temperature. The baseline characteristics of the two groups are shown in Table 1.

**Table 1:** Comparison of baseline variables between patients with confirmed COVID-19 and MP

Variables	COVID-19 (*n* = 10)	MP (*n* = 13)	*p* value
Age(years)	38.80 ± 13.43	34.46 ± 12.61	0.435
Sex (n, %)			0.580
Female	5 (50%)	5 (38.5%)	
Male	5 (50%)	8 (61.5%)	
BMI	24.85 ± 3.24	22.82 ± 4.54	0.245
Travel history (n, %)			0.560
Yes	2 (20%)	1 (7.7%)	
No	8 (80%)	12 (92.3%)	
COVID-19 contact history (n, %)			0.001
Yes	9 (90%)	1 (7.7%)	
No	1 (10%)	12 (92.3%)	
Temperature (°C)	37.55 ± 0.59	38.02 ± 1.04	0.215
LEU (× 10^9^ L)	5.95 ± 2.06	7.133 ± 3.04	0.303
LYM (× 10^9^ L)	1.43 ± 0.44	1.44 ± 0.50	0.279
NEU (× 10^9^ L)	3.55 ± 1.24	5.033 ± 2.74	0.127
MON (× 10^9^ L)	0.36 ± 0.33	0.54 ± 0.24	0.151
EOS (× 10^9^ L)	0.09 ± 0.04	0.59 ± 0.034	0.206
ESR (mm/h)	17.40 ± 5.13	20.92 ± 5.98	0.671
PCT (ng/L)	0.66 ± 0.21	0.14 ± 0.040	0.500
CRP (mg/L)	8.14 ± 4.47	25.94 ± 5.11	0.022

BMI, Body Mass Index; CRP, C-reactive protein; EOS, eosinophils; ESR, erythrocyte sedimentation rate; LEU, leucocytes; LYM, lymphocytes; MON, monocytes; MP, mycoplasma pneumonia; NEU, neutrophils; PCT, procalcitonin

The *p* values of the two groups were obtained using *t* tests for continuous variables and Fisher’s exact tests for categorical variables

### Changes in lobes involved and number of lesions

**Fig. 4:**
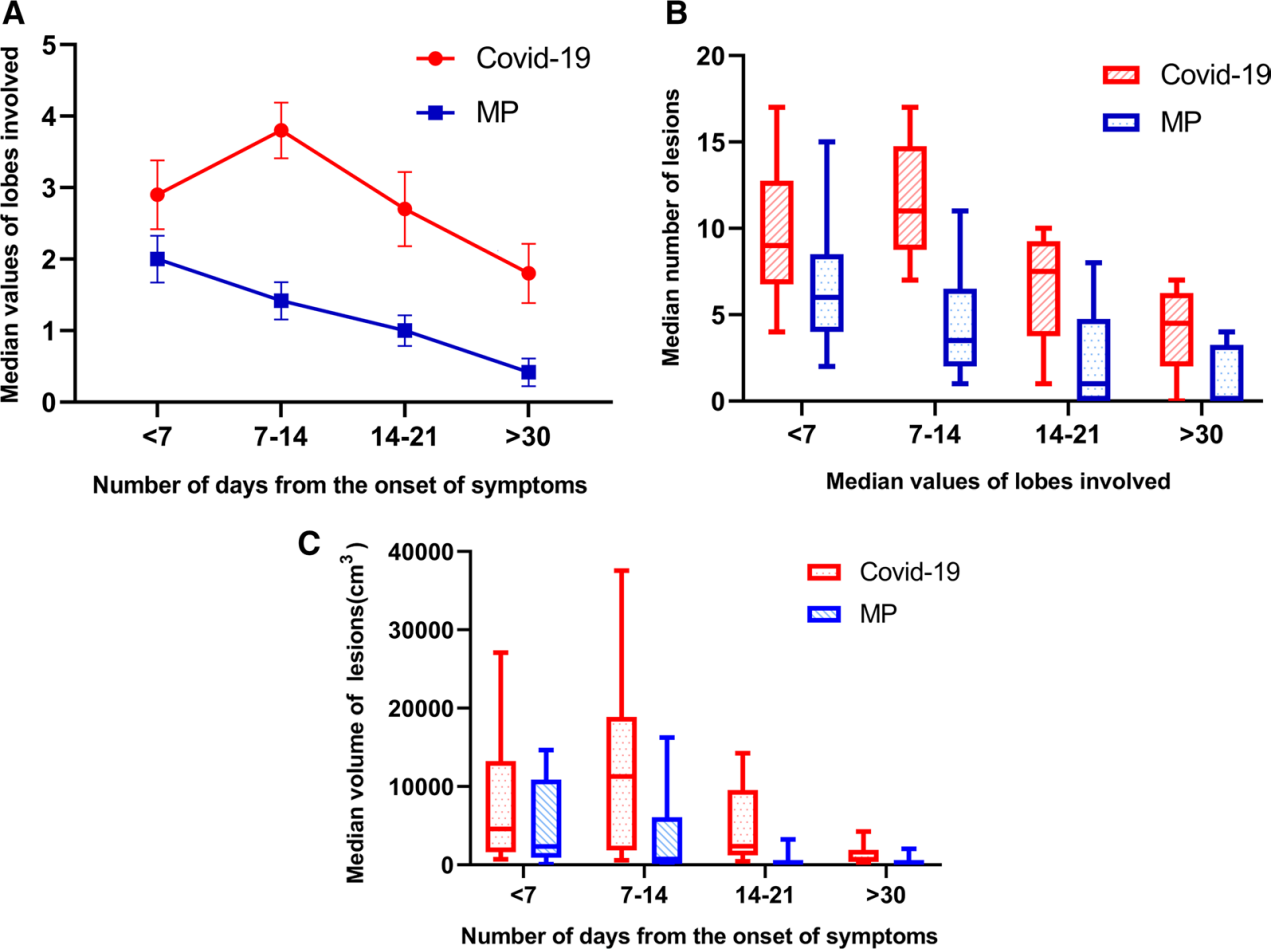
Quantitative changes in lung lesion volume. **A** Number of lobes involved. The lobes involved (**A**), number (**B**), and volume (**C**) of pneumonic lesions peaked within 7–14 days of symptom onset and then slowly decreased. Conversely, there were no peaks or declining trend over time in the MP group

Figure 4A shows the lesion changes in the lobes over time. After COVID-19 symptom onset, the involved lobes reached a peak within 7–14 days (median 3.8). In the MP group, there were two affected lobes on average within 7 days of symptom onset, and then gradually decreased, with no peak in the later period. The *p*-value between the two groups was 0.002, demonstrating a significant difference in the number of affected lobes between MP and COVID-19. Regarding the number of lesions over time, the average initial numbers were 9.7 and 6.42 (COVID-19 vs. MP, respectively). The number of lesions in COVID-19 group peaked in 7–14 days (average 11.70), but there was no peak in the MP group, and the number declined over time. The quantitative trends were significantly different (*p* < 0.01) (Fig. 4B).

### Quantitative changes in lung lesion volume

The results of the analysis of variance between the two groups (*p* < 0.01) indicated that the mean intragroup lesion volumes significantly changed over time. There was also a significantly difference in this parameter between the COVID-19 and MP groups (*p* = 0.043).

Compared with the first CT (first volume = 5446.46 cm^2^), lesion volume in the MP group was significantly decreased during follow-up. Of the 13 cases, 6 showed improvement and disappearance of lesions in the third examination. In the COVID-19 group, lesion volume increased during the second CT examination (first volume = 8098.48 cm^2^, second volume = 12,417.70 cm^2^), At the third inspection, the lesion volume began to decrease but was higher than that of the MP group (Fig. 4C). The total volumes of lesions between the two groups were significantly different (*p* = 0.021).

## Discussion

Although CT findings of bilateral GGO or consolidation may prompt radiologists to diagnose patients with COVID-19 [2, 3, 13, 14], there is a possibility of misdiagnosis based on imaging, because different diseases can show similar signs or findings. In adults, MP often manifests as non-specific interstitial changes (e.g., GGO and consolidation). Adult MP manifests as diffuse and/or multifocal ground-glass plaque lesions that can involve all lung lobes, which is similar to findings in viral interstitial pneumonia [4–6]. Therefore, it is important to distinguish between the two diseases. This is the first comparison between patients confirmed with mild COVID-19 and those suspected to have the disease but confirmed to have MP. We utilized quantitative image parameters that were automatically determined based on a deep learning algorithm to evaluate and compare longitudinal CT changes of COVID-19 and MP.

Compared with the first CT scan (< 7 days of symptom onset), the volume and number of lesions increased on the second CT scan (7–14 days) in the COVID-19 group then decreased slowly, which is consistent with reports in the literature of imaging findings peaking around 13 days after symptom onset [15–17]. In the MP group, lesion number, volume, and involved lobes gradually decreased after the first CT examination (< 7 days after symptom onset), and most lesions were absorbed by the third follow-up. In this study, nine MP cases were completely absorbed at the fourth follow-up, compared to just two COVID-19 patients with complete resolution at the 30-day follow-up. The residual signs at the final follow-up in both groups were mainly GGO, but more were observed in the COVID-19 group. The pathological absorption time was longer in COVID-19 compared to MP patients. The quantitative analysis using the Pneumonia-CT-LKM-PP model demonstrated that lung involvement with COVID-19 reached a peak 7–14 days after symptom onset, and this was the most prominent COVID-19 imaging change. The longitudinal change is conducive to distinguishing between COVID-19 and MP [16].

Patients with mild COVID-19 have a short interval between symptom onset and the first CT examination, and the patient’s lymphocyte count is still within the normal range, similar to the presentation of MP. Lymphocyte counts in both groups were normal and not significantly different from each other. In addition, the CRP level of the MP group was higher than that of the COVID-19 group, indicating that MP induced an obvious inflammatory response. This may be related to the enrollment criteria since COVID-19 patients had mild disease while the MP group did not.

The CT findings of 13 patients with MP showed GGO patterns distributed under the lung pleura with interlobular septum thickening. Combined with the history of fever and travel, they were suspected as having COVID-19 after the first CT examination. Although bronchial wall thickening has been reported in MP, this sign was not obvious in the MP group, and this intrinsic sign is also not specific. Combining quantitative CT changes and multiple nasopharyngeal rRT-PCR tests can ensure a clear diagnosis of COVID-19 pneumonia, but our experience is that when the viral pneumonia imaging manifestations appear, MP should be considered and specific IgM antibody detection should be performed if necessary.

Some limitations should be considered when interpreting our results. First, the sample size was small because we required three follow-up scans to longitudinally evaluate lesion changes. Second, this study was based on an open-source quantitative assessment model of pneumonia, which still requires the supervision of radiologists. Third, we did not analyze lung CT change patterns (e.g., GGO and consolidation) over time because it has been reported in the literature [3, 18–23].

In conclusion, the Pneumonia-CT-LKM-PP model based on deep learning algorithms can objectively and quantitatively evaluate imaging changes in COVID-19 pneumonia. Lesion number, volume, and the lobes involved reached their peaks within 7–14 days after symptom onset. These characteristics of COVID-19 may be used to distinguish it from a diagnosis of MP and evaluate treatment effects and prognosis. And serum mycoplasma IgM antibody detection is necessary to help differentiating the path when the viral pneumonia imaging manifestations appear.

## Data Availability

The datasets used and/or analyzed during the current study are available from the corresponding author on reasonable request.

## Abbreviations

COVID-19: Coronavirus disease-19
CRP: C-reactive protein
HRCT: High-resolution lung computed tomography
GGO: Ground glass opacity
MP: Mycoplasma pneumonia
rRT-PCR: Real-time reverse-transcriptase polymerase chain reaction
